# Hair loss due to Quetiapine usage : a case report

**DOI:** 10.1192/j.eurpsy.2023.1749

**Published:** 2023-07-19

**Authors:** A. Rhaouti, H. Boukidi, A. khallouk, F. Laboudi, O. Abderrazak

**Affiliations:** 1 Hôpital psychiatrique arrazi, salé, Morocco

## Abstract

**Introduction:**

Hair loss is a common clinical complaint, resulting from a wide variety of causes, a variety of medications prescribed to treat mental diseases may contribute to hair loss.

**Objectives:**

As far as we know, no previously published reports of alopecia associated with quetiapine were identified in Morocco.

Through this work we will try to expose the first case having this undesirable effect in our context, next to the rare cases in the literature.

**Methods:**

This article describes the experience of a 37-year-old male developing diffuse alopecia associated with use of quetiapin and recovering after cessation of the medicament is presented, besides presenting some rare cases that were found and described in other studies.

**Results:**

Among these psychotropic agents, this side effect is most often reported with the use of valproic acid and lithium It has been also reported with the atypical antipsychotic medicines olanzapine and risperidone, but only rare cases were reported in relation to quetiapine, In this paper, a 37-year-old male patient developing diffuse alopecia associated with use of quetiapine and recovering after cessation of the medicament is presented.

**Conclusions:**

The results of this study are the first known in morocco, establishing a relationship between hair loss and the use of quetiapine will be taken in consideration while prescriping this medicine. These results confirm the relatively new idea of the impact of quetiapine on hair loss, unlike older studies which suggested good tolerance of this molecule.

**Disclosure of Interest:**

None Declared

